# An immunohistochemical study of HER2 expression in primary brain tumors

**DOI:** 10.37796/2211-8039.1001

**Published:** 2020-03-28

**Authors:** Mazaher Ramezani, Shadi Siami, Mansour Rezaei, Sedigheh Khazaei, Masoud Sadeghi

**Affiliations:** a Molecular Pathology Research Center, Imam Reza Hospital, Kermanshah University of Medical Sciences, Kermanshah, Iran; b Students Research Committee, Kermanshah University of Medical Sciences, Kermanshah, Iran; c Departments of Biostatistics, Fertility and Infertility Research Center, Kermanshah University of Medical Sciences, Kermanshah, Iran; d Medical Biology Research Center, Kermanshah University of Medical Sciences, Kermanshah, Iran

**Keywords:** HER2, immunohistochemistry, primary brain tumor

## Abstract

**Background:**

Primary brain tumors (PBTs) include any tumor in the brain whose prognosis is weak because of their histological characteristics.

**Aim:**

Herein, this study aimed to assess the HER2 tumor marker frequency in PBTs.

**Materials and methods:**

This study was done on the samples of primary brain tumor diagnosis from 2008 to 2015.

**Results:**

Out of 107 patients of brain tumor that had a mean age of 40.4 years (61.7% men), the most common location of the tumor was in the supratentorial region (63.85% cases). The prevalence of high-grade astrocytoma (HGA) and low-grade astrocytoma (LGA) at diagnosis was 43.9% and 37.4%, respectively. With regard to HER2 score, HER2-positive (scores 2 & 3) was in 42.1% of patients. On the other hand, HER2-negative (−) was in 57.9%, 2*+* in 33.6%, and 3*+* in 8.4% of patients. The patients of LGA had significantly younger ages, lower HER2 positivity, and lower HER2 percent compared with the HGA patients.

**Conclusions:**

The type of brain tumors can impact on HER2 expression that high HER2 expression in HGA may be helpful for therapeutic aims. Further studies are required to support these results with a higher volume of patients in the world.

## 1. Introduction

Primary brain tumors (PBTs) involve any tumors in the brain that can start from different parts of the brain [1–3]. Their prognosis is poor because of their histologic features; nevertheless, few benign tumors are determined in areas of inoperable [4,5]. In the studies reported in the U.S. and Europe, the incidence rate of brain neoplasms varies between 17.6 and 22 per 100,000 persons [6]. Around 18,500 new cases of PBTs are distinguished every year in the USA [7]. A systematic review in Iran from 2000 to 2010 reported PBTs had a total incidence of 2.74/100,000 persons, the most usual histopathologies of which included meningioma, glioblastoma multiforme (GBM), astrocytoma and ependymoma [8]. Astrocytic tumors as a type of PBTs may progress to GBM, a very malignant tumor of the central nervous system (CNS) [9–14]. Medulloblastoma is the most frequent malignant brain tumor in children (a peak incidence at ages 5–7 years) [15–19], accounting for around 20% of all primary CNS tumors [16]. Recent studies have suggested that aberrations of the human epidermal growth factor receptor 2 (HER2) may be involved in astrocytic brain tumors [20–22]. HER2 is a significant prognostic target for metastatic breast carcinoma therapy [23,24]. HER2 overexpression is shown in a rate of 20%–40% in solid tumors and is usually correlated with a poor prognosis [25,26]. Trastuzumab (Herceptin) is the first anti-HER2 therapy, especially in some metastatic/ advanced solid tumors (breast and gastroesophageal cancers) [27,28] to be approved by the FDA for treatment of breast tumor [28–30]. The studies conducted in different areas on various ethnicities/races have indicated that HER2 can express in PBTs, whose expression depends on tumor location and tumor type [15,16,23,31–34]. Therefore, for the first time, we aimed to study the prevalence of HER2 marker in different types of PBTs in the West of Iran and Kurdish race.

## 2. Materials and methods

The Ethics Committee of Kermanshah University of Medical Sciences approved this study. This descriptive study was performed on the 107 samples diagnosed with the primary malignant brain tumor that they were received from the pathology laboratory of Imam Reza Hospital from 2008 to 2015.

### 2.1. Immunohistochemistry

**Fig. 1:**
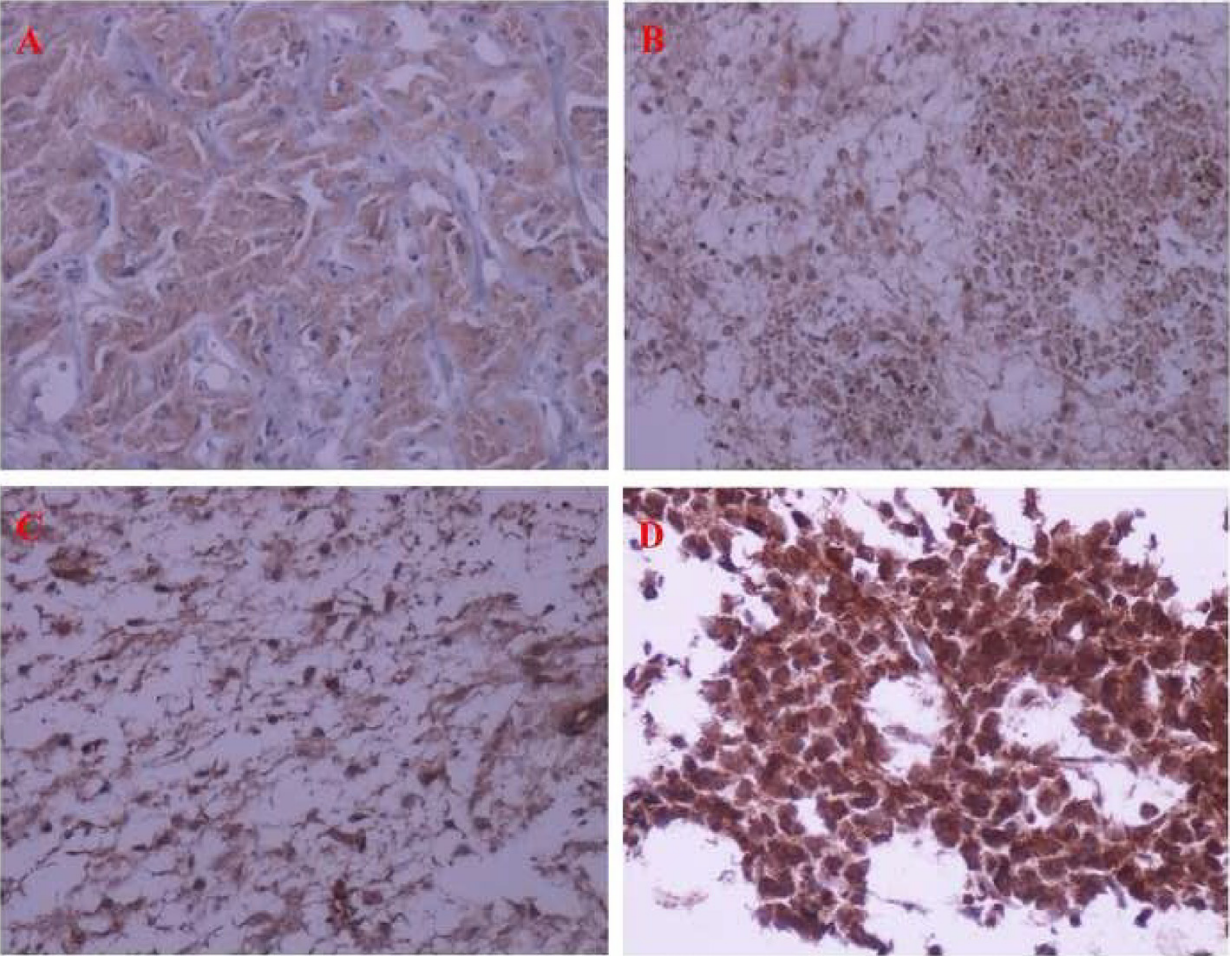
Immunohistochemistry staining of HER2, ×200 magnification: A) Pilocytic astrocytoma (score = 0), B) Pilocytic astrocytoma (score = 1), C) Pilocytic astrocytoma (score = 2), and D) Anaplastic astrocytoma (score = 3).

Formalin-fixed paraffin-embedded tissue (FFPET) specimens from each previously diagnosed PBT (based on the WHO classification) were cut into 4 micron-thick specimens and placed on glass slides. These specimens were stained by hematoxylin and eosin staining. The initial diagnosis was confirmed by the pathologist. Then, new specimens were provided, and immunohistochemistry (IHC) staining was done. Primary antihuman antibody against HER2 (c-erbB-2) oncoprotein (DAKO Diagnostics, Polyclonal Rabbit Anti-Human c-erbB-2 oncoprotein, Code A0485) was applied by the IHC method according to the manufacturer's brochure. Only staining of the cell membrane was considered specific for c-erbB-2 oncoprotein. Positive control samples were received from strong c-erbB-2 stained breast carcinomas and negative ones from normal breast tissue. Brown color in tumor cell cytoplasmic membrane was considered c-erbB-2- positive. The staining intensity was graded from 0 to 3+, as follows: no staining (0), low intensity and incomplete membrane staining (<10% of cells) (1+), low intensity and complete membrane staining (>10% of cells) (2+), and high intensity and complete membrane staining (>10% of cells) (3+). Tumors with scores 0 and 1+ were evaluated negatively, while those with scores 2+ and 3+ were evaluated positively [35]. Fig. 1 is shown IHC staining pattern of 0–3+ in pilocytic and anaplastic astrocytomas.

### 2.2. Statistical analysis

The data analysis was performed by SPSS version 16 software. The mean and standard deviation were computed for age; whereas, the number of patients and percentage were considered for the other data. Chi-square test was used for comparison of age between the factors and t-test for others among the variables.

### 2.3. Limitations

1. Losing the information from some patients.
2. Unavailability of some paraffin blocks.
3. A low number of cases in the Hospital.
4. Lack of fluorescence in situ hybridization (FISH) for HER2 (2+).

## 3. Results

Out of 107 patients with PBTs with a mean age at diagnosis of 40.4 years (range, 1–88 years), 61.7% of patients were male. Reporting tumor location in 85 patients, 74.1% of tumors occurred in the supratentorial region. The characteristics of the patients are shown in Table 1. The prevalence of high-grade astrocytoma (HGA) and low-grade astrocytoma (LGA) at diagnosis was 43.9% and 37.4%, respectively, primitive neuroectodermal tumor small round cell tumor lymphoma medulloblastoma (15.9%), mixed oligoastrocytoma (0.9%), hemangioblastoma (0.9%), and choroid plexus papilloma (0.9%). Checking HER2 scores, HER2-positive (scores 2 & 3) was in 42.1% of patients. On the other hand, HER2-negative (−) was in 57.9%, 2+ in 33.6%, and 3+ in 8.4% of patients.

Table 2 shows the correlation between some variables and HER2 status. There was no significant difference in age, sex, the tumor location, and the type of tumor between HER2-positive and HER2-negative patients.

The correlation between some variables and HER2 status demonstrated no significant difference in the variables (age, sex, the tumor location, and the type of tumor) between HER2 (−), HER2 (2+), and HER2 (3+) samples (Table 3).

**Fig. 2:**
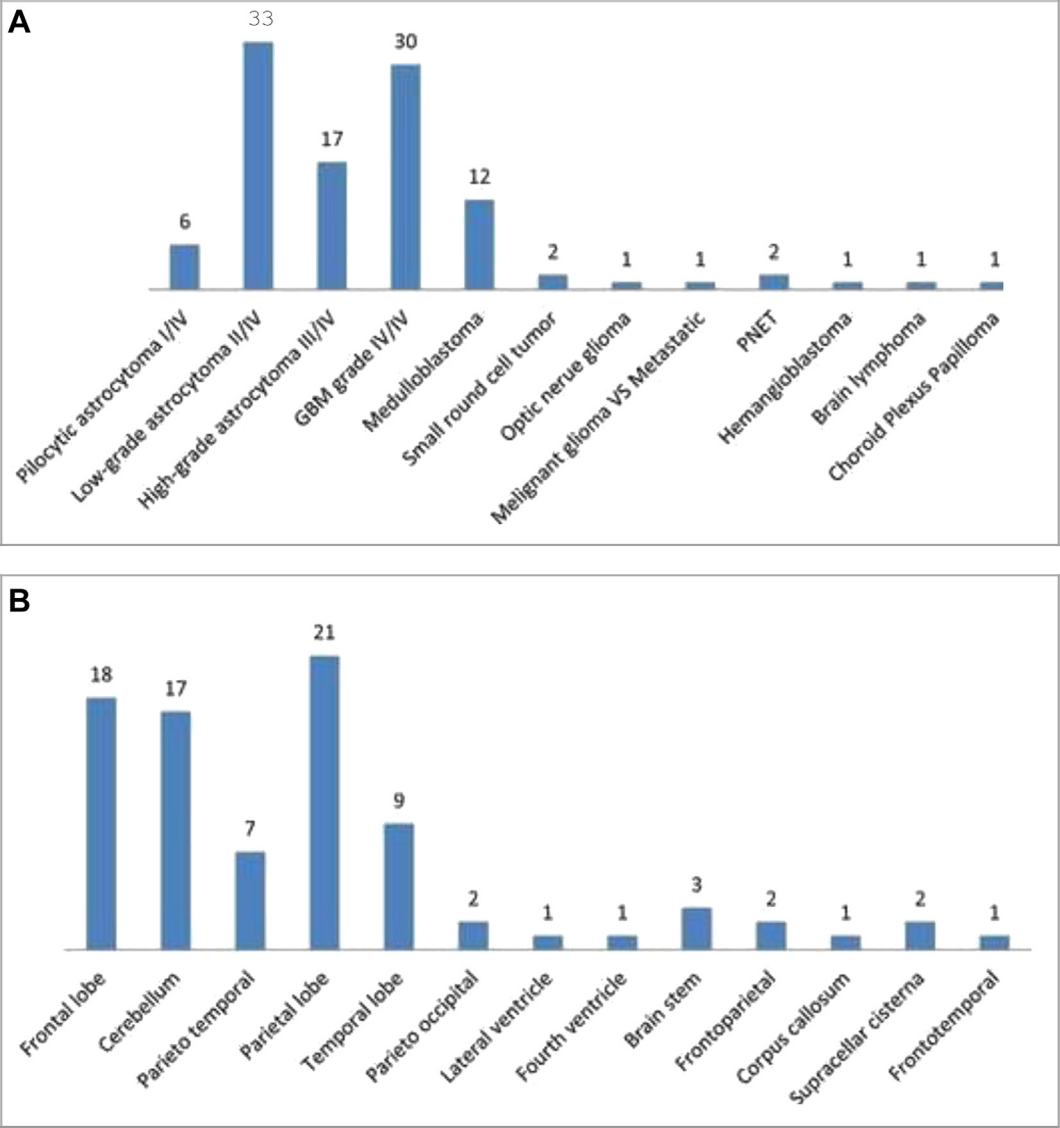
Prevalence of the patients with primary brain tumors, (A) subtypes and (B) tumor locations.

Fig. 2 illustrates the subtypes and tumor locations of PBTs more specifically. LGA ІІ/IV (33 patients) and GBM grade IV/IV (30 patients) had the highest prevalence. In addition, parietal lobe, frontal lobe, and cerebellum with 21, 18, and 17 patients, respectively, had the highest prevalence.

Comparing the correlation between some variables among LGA and HGA, there was a significant difference between the two astrocytomas in the variables of age, tumor location, HER2 status, and percentages (Table 4). The HGA patients had significantly an older age, higher HER2 positivity, and higher HER2 percentage compared with the LGA patients (P < 0.05). In addition, all HGA tumors were in the supratentorial region compared with 75% of LGA tumors in this location (P = 0.012).

## 4. Discussion

The present study analyzed HER2 expression in PBTs. The results showed that 42.1% of the patients were HER2-positive. In addition, 33.6% and 8.4% of tumors in the patients were HER2 2+ and 3+, respectively. In the comparison of LGA versus HGA, HER2 positivity in HGA tumors was significantly higher than LGA tumors.

One study [35] investigated seventy-two cases with LGA and HGA (56.9% GBMs, 13.9% diffuse astrocytomas, and 20.8% anaplastic astrocytomas) for HER2 overexpression, whose results showed 23.6% of patients were HER2-positive. There was no HER2 positivity in diffuse astrocytoma and pilocytic astrocytoma specimens, and overexpression was identified just for GBM subtype. In Reszeć’s study [9] on sixty-five patients with astrocytic tumors, including 17 diffuse astrocytomas, 23 anaplastic astrocytomas, and 25 GBMs, HER2 expression was observed in 88.3%, 88%, and 82.6% of diffuse astrocytoma, anaplastic astrocytoma, and GBM, respectively. In GBM, 11 samples (47.8%) were (+) positive and only 8 (34.8%) tumors were (++) positive. In 11/22 medulloblastoma tumors, 10%–50% of the tumors showed HER2 and HER4 positivity, which were detectable only in high-grade glial tumors [36]. Torp et al. [21] found HER2 positivity in 9 of 21 GBM tumors (43%).

A study [37] included 44 GBM patients (61.4% males) with a mean age of 79 years. All tumors were HER2 negativity by IHC and also amplification by FISH. Happasalo et al. [20] confirmed this result with astrocytic neoplasms. Meurer et al. [31] analyzed 40 medulloblastoma tumors and found that HER2 was positive in 23 patients (57.5%). In a retrospective study [32] on 57 and 16 cases with GBM and grade III gliomas, respectively, all GBM tumors expressed HER2 (2+ and 3+) highly and all secondary GBM tumors with low intensity (0 and 1+). A series of 70 cases with childhood medulloblastoma were analyzed for HER2 expression by IHC, sixty of which (85.7%) were found to be positive in IHC analysis. Ahmed et al. [15] showed a 40% HER2 expression in medulloblastomas. Out of 149 GBM cases (54.4% males and the mean age of 64 years); HER2 overexpression was found in 23 cases (15.4%) [23]. Potti et al. [38] checked 347 adult patients (the mean age of 53 years with 55.6% males) with PBTs. It was detected that 10.4% of the archival pathologic samples identified the presence of HER2 overexpression by the IHC.

Gulati et al. [33] investigated HER2 expression in 31 cases with anaplastic astrocytomas with three monoclonal antibodies, including CB11, 3B5, and 5A2. HER2 positivity was observed in 45%, 100%, and 52% of cases, respectively. This discrepancy in results may be due to using different monoclonal antibodies and different interpretations of the positive samples. One study [32] showed better survival with low HER2 expression in patients with medulloblastoma tumors. Koka et al. [23], after age, smoking history, performance status, and treatment adjusting of GBM patients, revealed that HER2 overexpression significantly raised the early mortality odds (the median survival of patients for HER2 overexpression was four months compared to eight months for lack of overexpression), which were confirmed by Potti et al. [38] Therefore, the results showed that overexpression of HER2 may be a weak prognostic marker in GBM cases [23] and astrocytic tumors of the brain [36].

The studies suggested that HER2 expression may be included in the development and progression of astrocytic brain tumors and may be potentially important due to the role of Herceptin therapy in these tumors [16,39,40].

## 5. Conclusions

Considering the high expression of HER2 in most brain tumors, overexpression of HER2 may be a weak prognostic marker in patients with PBTs. But the results showed the type of brain tumors can impact on HER2 expression and high expression in HGA may be helpful for therapeutic aims. Further studies are recommended to be conducted on a more number of patients in various areas to confirm these results.

## Figures and Tables

**Table 1:** Baseline characteristics of the patients with primary brain tumors (n = 107).

Variable	N (%)	Variable	N (%)
**Age**, year		**HER2 score**	
Mean ± SD	40.4 ± 22.35	0	6 (5.6)
Range	1–88	1	56 (52.3)
		2	36 (33.6)
		3	9 (8.4)
**Sex**		**HER2 status**	
Male	66 (61.7)	Negative	62 (57.9)
Female	41 (38.3)	Positive	45 (42.1)
**Tumor location**		**HER2**, %	
Sellar	2 (2.4)	Mean ± SD	55.31 ± 28.15
Supratentorial	63 (74.1)	Range	0–100
Brain stem	3 (3.5)		
Cerebellum	17 (20)		
NA	22		
**Complaints**		**HER2**, %	
Lateralized	18 (30)	≤10 (−)	6 (5.6)
Generalized	22 (36.7)	11-50 (+)	43 (40.2)
Mixed	20 (33.3)	≥51 (**++**)	58 (54.2)
NA	47		
**Tumor type**			
LGA	40 (37.4)		
HGA	47 (43.9)		
Mixed oligoastrocytoma	1 (0.9)		
PNET-SRCTLM	17 (15.9)		
Hemangioblastoma	1 (0.9)		
Choroid plexus papilloma	1 (0.9)		

**Abbreviations**: PNET-SRCTLM, Primitive neuroectodermal tumor small round cell tumor lymphoma medulloblastoma; LGA, Low-grade astrocytoma; HGA, High-grade astrocytoma; SD, Standard deviation; NA, Not available.

**Table 2:** The correlation between variables and HER2 status in the brain tumor patients (n = 107).

Variable	HER2-positive	HER2-negative	*P*-value
**Age**, year			
Mean ± SD	41.44 ± 22.89	39.02 ± 22.09	0.582
**Sex**			
Male	27 (60%)	39 (62.9%)	0.458
Female	18 (40%)	23 (27.1%)	
**Tumor location** (n = 85)			
Sellar	1 (2.6%)	1 (2.2%)	
Supratentorial	30 (76.9%)	33 (71.7%)	
Brain stem	1 (2.6%)	2 (4.3%)	
Cerebellum	7 (17.9%)	10 (21.7%)	0.934
**Tumor type**			
LGA	12 (26.7)	28 (45.2%)	
HGA	25 (55.6%)	22 (35.5%)	
Mixed oligoastrocytoma	0 (0%)	1 (1.6%)	
PNET- SRCTLM	7 (15.6%)	10 (16.1%)	0.179
Hemangioblastoma	0 (0%)	1 (1.6%)	
Choroid plexus papilloma	1 (2.2%)	0 (0%)	

**Abbreviation**: PNET-SRCTLM, primitive neuroectodermal tumor small round cell tumor lymphoma medulloblastoma; LGA, Low-grade astrocytoma; HGA, High-grade astrocytoma; SD, Standard deviation.

**Table 3:** Correlation between study variables and HER2 status in the brain tumor patients (n = 107).

Variable	HER2 (−)	HER2 (2+)	HER2 (3+)	*P*-value
**Age**, year				0.843
Mean ± SD	39.0 ± 22.1	41.1 ± 20.8	42.8 ± 31.2	
**Sex**	39 (62.9%)	24 (66.7%)	6 (66.7%)	0.176
Male Female	23 (37.1%)	12 (33.3%)	3 (33.3%)	
**Tumor location** (n = 85)				0.865
Sellar	1(2.2%)	1 (3.3%)	0 (0%)	
Supratentorial	33 (71.7%)	24 (80%)	6 (66.7%)	
Brain stem	2 (4.3%)	1 (3.3%)	0 (0%)	
Cerebellum	10 (21.7%)	4 (13.3%)	3 (33.3%)	
**Tumor type**				0.052
LGA	28 (45.2%)	11 (30.6%)	1 (11.1%)	
HGA	22 (35.5%)	20 (55.6%)	5 (55.6%)	
Mixed oligoastrocytoma	1 (1.6%)	0 (0%)	0 (0%)	
PNET- SRCTLM	10 (16.1%)	5 (13.8%)	2 (22.2%)	
Hemangioblastoma	1 (1.6%)	0 (0%)	0 (0%)	
Choroid plexus papilloma	0 (0%)	0 (0%)	1 (11.1%)	

**Abbreviation**: PNET-SRCTLM, primitive neuroectodermal tumor small round cell tumor lymphoma medulloblastoma, LGA, Low-grade astrocytoma; HGA, High-grade astrocytoma; SD, Standard deviation.

**Table 4:** Correlation between study variables and astrocytic tumors in the brain tumor patients (n = 87).

Variable	Low-grade astrocytoma	High-grade astrocytoma	*P*-value
**Age**, year			<**0.001**
Mean ± SD	35.6 ± 20.6	50.8 ± 16.2	
**Sex**	24 (60%)	31 (66%)	0.362
Male			
Female	16 (40%)	16 (34%)	
**Tumor location** (n = 67)	2 (7.1%)	0 (0%)	**0.012**
Sellar			
Supratentorial	21 (75%)	39 (100%)	
Brain stem	1 (3.6%)	0 (0%)	
Cerebellum	4 (14.3%)	0 (0%)	
**Her2 status**	28 (70%)	22 (46.8%)	**0.024**
Negative			
Positive	12 (30%)	25 (53.2%)	
**HER2**, %	42.4 ± 28.1	65.4 ± 24.6	<**0.001**
Mean ± SD			
Range	0–100	5–100	
**HER2, %**	4 (10%)	1 (2.1%)	**0.006**
≤10 (−)			
11–50 (**+**)	22 (55%)	14 (29.8%)	
≥51 (**++**)	14 (35%)	32 (68.1%)	

**Abbreviation:** SD, Standard deviation.
